# ERRATUM

**DOI:** 10.1590/0034-7167.20247701e02

**Published:** 2024-02-26

In the article “Active teaching model to promote critical thinking”, with DOI number: https://doi.org/10.1590/0034-7167-2018-0002, published in Revista Brasileira de Enfermagem, 2019;72(1):293-8, on page 297:

Include before REFERENCES:

**FUNDING**

This study was financed in part by the Coordenação de Aperfeiçoamento de Pessoal de Nível Superior - Brasil (CAPES) - Finance Code 001.

